# A qualitative study of the maternal health information‐seeking behaviour of women of reproductive age in Mpwapwa district, Tanzania

**DOI:** 10.1111/hir.12329

**Published:** 2020-10-14

**Authors:** Mohamed Kassim

**Affiliations:** ^1^ Department of Information Studies University Library University of Dar es Salaam Dar es Salaam Tanzania

**Keywords:** Africa, east, demography, focus groups, information need, information‐seeking behaviour, midwifery, women's health

## Abstract

**Background:**

Active engagement in seeking maternal health information among women of reproductive age is vital in promoting positive health‐seeking behaviour and improving maternal health outcomes.

**Objective:**

This study aimed to explore maternal health information‐seeking behaviour of women of reproductive age in a rural Tanzania.

**Methods:**

Using a qualitative research approach, the study held eight focus group discussions to collect data from a group of purposively selected women respondents. Data were analysed through thematic analysis.

**Results:**

Women need a range of maternal health information for their informed health decision making. However, while they indicated the need to seek that information from professional health providers, they received most of it from non‐professional and informal sources, including community health workers, traditional birth attendants and their family members. The use of these sources is attributed to, among other factors, unavailability of health facilities and limited access to professional health care.

**Conclusion:**

Access to relevant and reliable maternal health information is essential in creating awareness and empowering women to make informed decisions about their reproductive health. Women’s use of informal sources to meet their various information needs increases the chances of receiving unreliable information that may result in coming up with poor decisions making.

**Key messages**

In some rural areas in countries such as Tanzania, women need better health information to make informed decisions about their pregnancy and the health care of their infants.
Community health workers and traditional birth attendants need more training to provide women with the information they need.
Evaluation of rural maternal health services should take careful account of the views of the women for whom the services are intended.

## Introduction

Although there is a global decline of maternal mortality by 38% from 451,000 in the year 2000 to 295,000 deaths in 2017 as indicated in the World Health Organization’s (WHO) report (WHO, 2017), this problem remains a major challenge to many health systems worldwide. Many women continue to die due to factors associated with pregnancy and/or delivery complications. This problem, however, is more prevalent in developing countries than in developed countries as evidence from studies (Black, Laxminarayan, Temmerman, & Walker, 2016; WHO, 2017) shows that while less than 6% of these deaths occur in developed countries, a majority of them (94%) occur in developing countries, including Tanzania. As such, the need to improve maternal health and thus reduce maternal mortality has been a major focus at both global and national levels.

In Tanzania, the need to improve maternal health is reflected in the National Health Policy by the Ministry of Health, Community Development, Gender, Elderly and Children (MoHCDGEC) in which the government has expressed commitment to provide free maternal and child health care services to increase access for pregnant women (MoHCDGEC, 2017). Furthermore, various policies, strategies and interventions have been implemented to improve the provision of maternal health care services at all levels. These include the adoption of Safe Motherhood Initiative (SMI) – 1989; the National Strategy for Growth and Poverty Reduction (NSGPR) (United Republic of Tanzania [URT], 2005; United Republic of Tanzania [URT], 2010); Health Sector Strategic Plan (Ministry of Health and Social Welfare [MoHSW], 2009); Reproductive and Child Health Strategy (2005 – 2010); National Road Map Strategic Plan to Accelerate the Reduction of Maternal and Newborn Mortality (MoHSW, 2008, 2014); Millennium Development Goal 5 (MDG) of reducing maternal mortality by three‐quarters by 2015 (133 maternal deaths per 100,000 live births) (MoHSW, 2008); and the adoption of Sustainable Development Goals (SDGs) of reducing maternal mortality to less than 70 per 100,000 live births by 2030 (United Nations [UN], 2015; URT, 2013).

As a result of these efforts, the country has witnessed a decline in maternal mortality from 910 maternal deaths per 100,000 live births in 1990 to 410 in 2013, a change considered as progress in addressing this problem (WHO et al., 2014). Nevertheless, this progress has not been sufficient considering that it did not reach the country’s requirements to achieve the MDG 5 of reducing maternal deaths by 75% by the end of 2015 (Afnan‐Holmes et al., 2015; Nieburg, 2012). Furthermore, evidence from maternal health reports shows that there has been an increase in maternal deaths with a ratio of 556 deaths per every 100,000 live births by 2015 (MoHCDGEC et al., 2016) thus suggesting that maternal mortality is still a challenge in the country. The risk of maternal death in Tanzania according to the National Bureau of Statistics (NBS) and Office of Chief Government Statistics (OCGS) (2015) is high in rural areas where it is estimated that 3 women per 1,000 are likely to die compared to 2 per every 1,000 women in urban areas.

Most maternal deaths in Tanzania are caused by factors attributed to low uptake of maternal clinics during pregnancy, childbirth, low awareness of danger signs of obstetric complications, poor access to reproductive services and weak health system (Shija, Msovela, & Mboera, 2011). Other causes of maternal deaths include haemorrhage, eclampsia, HIV, malaria, long distance to health facilities (Hanson et al., 2015; Laddunuri, 2013), obstructed labour and unsafe abortion (Laddunuri, 2013). Other studies, particularly Killewo et al. (2006) have associated maternal deaths with delays in making quality health decisions to seek care and delays in accessing emergency obstetric care during life‐threatening obstetric complications. These deaths are also associated with poor family planning which results in every woman giving birth to 5 or 6 children on average, where 1 in 3 women begin childbearing before reaching 18 years of age, thus getting exposed to more maternal and neonatal mortality risks (UNICEF, 2012).

Understandably, there is no single approach to addressing this problem (Das, 2013; Hanson et al., 2015). Provision of timely and relevant maternal health information to help pregnant women make informed choices and decisions can also contribute to the reduction of both maternal morbidity and mortality. One of the expected influences of such information is increasing awareness of the need to utilise maternal health care services (ante and post‐natal care) among mothers. Active involvement in seeking maternal health information has been linked to the promotion of positive health behaviour among women, thus improving their maternal health outcomes (Das, 2013; Shieh, Broome, & Stump, 2010).

Evidence from previous studies (Collins, 2007; Greene et al., 2013) shows that with accurate maternal health information, pregnant women are more likely to make informed quality reproductive health choices and decisions. As a result, the women can prevent complications and abnormalities during pregnancy, seek and utilise available maternal health care services on time, and have positive perceptions of both pregnancy and childbirth. In particular, the reduction of delays in seeking appropriate care during pregnancy can tremendously reduce instances of both maternal morbidity and mortality.

Generally, the value of access to timely, relevant and affordable maternal health information to helping women of reproductive age to make prompt informed decisions about their maternal health is clear. Surprisingly, maternal health information needs and seeking behaviour of these women, particularly those living in rural Tanzania have not been well explored. This study, therefore, aimed to explore the maternal health information‐seeking behaviour of women of reproductive age in Mpwapwa district, rural Tanzania. The specific objectives of the study were twofold: to identify the maternal health information needs of the women of reproductive age and to find out the challenges the women face when seeking that information.

## Methods

This study was conducted in Mpwapwa district, Dodoma region, central Tanzania. This district had a relatively high maternal mortality rate in the country (Urassa, Pembe, & Mganga, 2012). According to Dodoma Regional Commission's (DRC) report (DRC, 2014), in 2010 the region had a total of 85 cases of maternal deaths with Mpwapwa district having the highest maternal deaths (18 cases) compared to other districts in the region.

The study employed an exploratory descriptive qualitative research design to investigate maternal health information needs and seeking behaviour of women of reproductive age in the area. The study involved women of reproductive ages from 15 to 49 years. Purposive sampling was used to select respondents from four wards namely Mpwapwa town, Ving’hawe, Lupeta and Mazae.

Focus group discussions (FGDs) were used to collect data for this study. A total of eight FGDs, two in each selected ward, were held. Each FGD comprised of 8 respondents thus making a total of 64 participants in all FGDs. Having a maximum number of 8 participants in each FGD was considered ideal since it makes it possible to sustain a discussion and thus gain a diversity of perspectives from the participants (Babbie, 2008). Data from the respondents were analysed through thematic analysis using QSR Nvivo 10. Audio recordings from focus group discussions were transcribed, and from the written transcripts, key themes were identified and highlighted. Different themes that emerged were reviewed and those which were similar were grouped.

## Results

### Maternal health information needs

Findings from the study reveal that majority of the respondents needed different types of maternal health information that could help them in their informed reproductive decision making processes. One of the maternal health information found to be needed is information related to the early signs of pregnancy. During the discussions, the women revealed that it took some time for some of them, particularly first‐time mothers, to realise that they were pregnant. This was because they were not aware of the early signs of pregnancy. Other respondents mentioned that they confused early signs of pregnancy such as nausea, fatigue and vomiting with symptoms of malaria. Because of this, the women started attending the clinic for antenatal care very late. One respondent, for instance, pointed out that:

I think information about early signs of pregnancy is very important to us first time‐mothers. It took a while for me to realize that I am pregnant. At first, I thought it was just malaria as I was getting tired and vomiting. I even tried to take some malaria pills believing that they will help. I only came to know that I am pregnant after explaining my situation to my elder sister who then suggested that I should try to take a pregnancy test (FGD participant, Mangang’u village, August 2017).

The women also expressed the need to be provided with information related to danger signs of pregnancy. For instance, during the discussion, in a number of the FGDs, some women mentioned that when they first missed their menstrual period, they thought that they were pregnant. However, after some time, they started seeing some blood spots and thus thought that they have started bleeding again, and therefore, they were not pregnant. Describing the situation, one participant stated that:

I remember having some problems with my first pregnancy. When I first missed my periods, I thanked God thinking that I am pregnant. But as time went on, I started bleeding again…I was very scared and confused. However, after several months, the bleeding stopped. Though I don’t know what exactly caused the stop, I thanked God that my pregnancy was still there, and I was able to deliver (FGD participant, Iyoma village, August 2017).

Respondents have also been found to need information related to overdue pregnancy. The need for this type of information was because there were many misconceptions about it. For example, while some of the respondents associated this problem with other pregnancy‐related complications, others associated it with witchcraft which was aimed at preventing an expectant mother from delivering. One woman pointed out that:

…it is very strange that some pregnant women exceeded the normal gestational period of nine months…for instance, others may even go up to ten or twelve months. What causes this…we do not know. Some women even think that they have been bewitched (FGD participant, Kisokwe village, August 2017).

The results also show that women also need information on obstetric fistula. The results show that there were also many misconceptions about obstetric fistula among the study respondents as some of them associated it with witchcraft. Specifically, the women need to be informed about the causes of obstetric fistula, possible treatment, and what they should do to prevent it. They pointed out that fistula‐related information is very important to them since it will help them to be more aware of it and thus know what to do in case someone has it. Explaining the need and importance of this information, one woman stated that:

One of the problems that I think we need to be provided with information about is the uncontrolled passing of urine, especially after delivery. I have seen some women in my village who have been isolated from people because of the smell that comes from their uncontrolled urine. It is important that we know why this happens and if possible be informed of what to do so that we do not get it (FGD participant, Idilo village, August 2017).

The women were also interested in knowing if it is okay for them to use traditional medicines during pregnancy. They associated their need to use traditional medicines with the unavailability of modern health facilities in their villages. They wanted to be sure about the safety of these medicines if used during pregnancy. In one of the FGDs, one participant said:

The problem of unavailability of a health facility in our village has made it difficult to use modern medicines. As such, many of us are compelled to rely on traditional medicines as our primary healthcare. However, the problem is that we don’t know exactly the side effects of these medicines especially when they are used by a pregnant woman (FGD participant, Kisokwe village, August 2017).

Information on immunisation and prevention of communicable diseases has also been reported to be highly needed by the respondents. The women specifically mentioned that they want to be provided with malaria and HIV/AIDS‐related information. In the discussions, participants stated that they need this type of information so that they can protect themselves and their children from these diseases. Explaining the need for this information, one respondent said:

I would like to be provided with information on how to protect my baby from transmitted diseases, especially malaria…most children in our village are easily infected by this disease. This information will help us to address this problem (FGD participant, Makutupa village, August 2017).

During the discussions, it was obvious that women also need to be provided with information related to breastfeeding and introduction of solid foods to their children. The study reveals that some of the women introduced solid foods to their children less than the recommended six (6) months period of exclusive breastfeeding. A participant in one of the FGDs pointed out that:

I would like to know exactly when we are supposed to introduce other types of food to our children. Some say it is six months, but what I know is that other women give their children solid food even when they are three months old. What harm will these foods have if given to children before they are six months old? (FGD participant, Bumila village, August 2017).

Findings from the study further indicate that women respondents need information related to exercising during pregnancy. The women stated that apart from being told to engage in physical exercises during pregnancy, no further information was provided about the types of exercises they are supposed to engage in and the frequency of doing so. For that reason, the women expressed interest in the types of exercises that are recommended for pregnant women to ensure the safety of both the mother and her unborn child. One woman stated that:

Sometimes when I go to the clinic the nurses tell us to do some exercise when we get home. But what they don’t tell us is the types of exercises and to what extent we are supposed to do those exercises. I am worried about the safety of my child. (FGD participant, Mangang’u village, August 2017).

Furthermore, the findings reveal that women are in need of family planning information. During the discussions, women pointed out that this type of information is inadequately provided, especially in areas where health care facilities are not available. As a result, there are a lot of misconceptions about family planning among women. Commenting on the matter, one woman stated that:

We need more clarification on family planning. You know, when we meet as women in our groups, you hear a lot about family planning. Some may say it has side effects…some may say it doesn’t. We are in a dilemma. That is why you may find that some people are using while others are not (FGD participant, Bumila village, August 2017).

### Barriers encountered by women when seeking maternal health information

The study findings have revealed that women are faced with several barriers that deterred them from seeking maternal health information. Respondents mentioned that because of the shortage of health facilities in their villages, they are compelled to travel long distances to where these facilities are available so that they can have access to professional health care and maternal health information. This, according to the women, not only discourages them from seeking maternal health information from skilled health care providers but also affects their utilisation of professional health care. Responses obtained reveal that the women received most of their maternal health information from non‐professional health care workers, particularly community health workers (CHWs) and traditional birth attendants (TBAs). They also mentioned that they get some information from their immediate family members, namely their mothers, grandmothers, mothers‐in‐law, their spouses and other female relatives. One respondent said:

The clinic is very far from here…that means you need to be well prepared for you to go there. Sometimes you spend the whole day only attending the clinic. This has even led to poor utilization of maternal healthcare services as many of us now turn to our female relatives for maternal health information (FGD participant, Iyoma village, August 2017).

Respondents are also concerned about the quality of health services offered through outreach programs. During the discussions, participants mentioned challenges such as inadequate time allocated to these programs, small numbers of skilled health care providers against the large numbers of women expected to receive these services, and lack of privacy. As such, although it is some of the women’s wish to access this information from skilled health care providers, they are compelled to seek information from non‐skilled health care providers. Contributing on this, one participant shared the following:

The problem with outreach programs is that most of them are very crowded. As such, the available health workers can't provide maternal health education to all the women attending these programs. Sometimes it is even difficult for us to understand the information provided especially when there is a big crowd with no privacy (FGD participant, Mangang’u village, August 2017).

Although the respondents admitted to receiving maternal health information from CHWs, they are sceptical about the level of knowledge of these health workers on various maternal health issues. It was revealed during the discussion that most women in the studied communities feel that CHWs have inadequate knowledge of various maternal health issues. The women pointed out that, unlike skilled health providers, most of the CHWs have basic knowledge on maternal issues and thus not reliable sources of maternal health information. One participant said:

Even though we seek information from community health workers, we are still worried about the credibility of the information they give us. We understand that some of them have received training about maternal health, but the training is only for a few weeks. That does not make them experts in maternal health. It is just that we do not have other reliable sources of maternal health information (FGD participant, Ving’hawe village, August 2017).

Findings also show that there is a problem of male dominance over decision making. It was revealed during the discussion that in most of the studied communities, most family decisions including those related to maternal health are solely made by men. The women pointed out that although they are the ones carrying the pregnancy, the power to decide when to seek care and where to deliver is entirely in their spouses' hands. As a result, women are not able to make their own decisions even when their spouses are not around. Commenting on the matter, one woman said that:

…because of our traditions, some of us are not allowed to get out of the house and go to the hospital without permission from our husbands. This is all because of the patriarchal system which does not allow women to make decisions on their own. It is the husband who decides for his wife… (FGD participant, Kisokwe village, August 2017).

In relation to the aforementioned challenge, women also revealed that there is either limited or poor male support for various maternal health issues. It was reported in the discussions, for instance, that some men are reluctant to accompany their pregnant partners to the hospital for antenatal care. As a result, some pregnant women are compelled to request other men to accompany them to the hospital, especially during the first antenatal visit when they are supposed to go with their partners. Explaining this, one woman stated that:

Unfortunately, our partners are refusing to accompany us to the hospital for antenatal care. For instance, my husband used to say, ‘it is you who is carrying the pregnancy, why should I go with you to the clinic?’ Yet, when you go there alone you are denied service unless you bring your partner along. This is very discouraging (FGD participant, Bumila village, August 2017).

Poor transport and communication infrastructure facilities were also mentioned by women as a challenge to their seeking of maternal health information. During the discussions, the women complained about the absence of tarmac roads in their villages. It was pointed out that due to poor road infrastructure, the only means of transport affordable to most of the women and their families is ‘*boda‐boda’* (motorcycle). However, because of the nature of the roads, it is very difficult for pregnant women to use such means of transport as it risks both the life of the mother and that of the foetus. As a result, many women fail to attend antenatal care services in areas where health care facilities are available. Expressing her concern, one participant said:

How do you expect me to use a bodaboda to travel such a long distance with this type of road? With this big belly…do you want me and my child to die on the road? The road from here to the district hospital is very rough and the problem is worse during rainy seasons. It is not that we don’t want to go to the district hospital; it is the prevailing circumstances that force us not to (FGD participant, Bumila village, August 2017).

Low level of education among pregnant women and their male partners has been found to affect the information‐seeking behaviour of the women in the study area. Respondents mentioned that they sometimes face difficulties in using written sources of information because of their low levels of education. It was reported, for example, that due to illiteracy, some women fail to read and understand the maternal health information provided in print format. One respondent shared the following:

Sometimes the nurses at the clinic give us some documents (brochures) and tell us to read when we get home. The problem with me is that I have never been to school, so it is difficult for me to understand what is written in these documents (FGD participant, Kisokwe village, August 2017).

Furthermore, the women attributed their male partners’ unwillingness to support them on various maternal health issues to low levels of education. The women claimed that if their partners were educated, they would be willing to support their spouses in all matters related to maternal health, including accompanying them to clinics for antenatal care.

Family responsibilities were also reported to affect women’s information‐seeking behaviour. It was revealed during the discussion that women's engagement in various household activities including taking care of their families denied them adequate time to engage in health information‐seeking activities. In particular, the women lamented the scarcity of water in their villages which compels them to spend too much of their time fetching water from nearby villages. Describing this situation, one woman stated that:

Most of the areas in our villages are faced with the problem of shortage of water. As such, we spend most of our time fetching water from nearby villages. This, together with other family responsibilities such as cooking and taking care of the other children deny us time to attend clinics where we can get maternal health information (FGD participant, Bumila village, August 2017).

## Discussion

This study has focused on maternal health information needs and seeking behaviour of women of reproductive age in a rural Tanzania. Findings from the study indicate that there are various unmet needs of maternal health information in almost all the studied villages. The fact that respondents, particularly first‐time mothers, confused early signs of pregnancy with some symptoms of malaria namely nausea, vomiting and fatigue suggests that there is low awareness of early signs of pregnancy among them. However, this confusion may be because malaria is very common in the studied communities as reported in previous studies (Mboera et al., 2008; Shemdoe, 2011). Moreover, it is apparent from the findings that there is low awareness of various danger signs of pregnancy among women in the studied villages. Studies have associated low awareness of danger signs of pregnancy with delays to make decisions to seek professional health care (Kabakyenga et al., 2011) and increased maternal morbidity and mortality among pregnant women (Pembe et al., 2009). Therefore, apart from suggesting that the women in this area are aware of the importance of this type of information, this indicates that the need for this information must be addressed. Low awareness of the danger signs of pregnancy among women is also reported in a rural coastal area of Tanzania (Pembe et al., 2009).

Furthermore, it is clear from the findings that the misconceptions among most women in the studied communities about overdue pregnancies, obstetric fistula and family planning are due to an acute shortage of information about the same. The fact that women associate the problems of overdue pregnancy and obstetric fistula with witchcraft suggests a necessity to provide them with adequate information to help them become aware of these signs of pregnancy complications that contribute to perinatal deaths as reported in previous studies (Aborigo et al., 2014; Bakar, Mmbaga, Nielsen, & Manongi, 2019). Shortage of this important information may lead women to continue associating these problems with witchcraft, something which may affect their utilisation of available maternal health care services. Additionally, the misconception about family planning suggests that women in the studied villages have inadequate knowledge about various types of family planning methods. A shortage of this information may result in low or no usage of various family planning methods among women and their male partners thus increasing the risk of maternal morbidity and mortality for pregnant women.

Findings from the present study also suggest that although women wish to receive maternal health information from professional health care providers, they received most of this information from informal sources. Consistent with findings from previous studies (Mwangakala, 2016; Silali & Owino, 2016), this study also found that challenges such as long distances to health facilities and poor maternal health services from outreach programs compel the women to rely on informal sources to meet their maternal health information needs. It can, therefore, be concluded from these findings that it is not always that people just decide to seek health information from non‐credible sources; rather, they are mostly compelled to do so by the inaccessibility of reliable sources such as professional health care providers.

Although the role of informal sources of health information cannot be overemphasised in areas with limited access to professional health care, other studies have criticised their use due to their limited knowledge on human health (Malata & Chirwa, 2011; Song et al., 2013). While other informal sources do not have any formal training on various maternal health issues, CHWs normally receive training although it is less than what professional health care workers get. However, this training does not make them as authoritative sources of maternal health information in areas where there is limited access to professional health care. Therefore, the findings in this study underline the importance of professional health care providers as viable sources of maternal health information since they have adequate knowledge and expertise on various maternal health issues.

The findings of this study further show that respondents face several other challenges that influence their maternal health information‐seeking behaviour. Male dominance of decision making and their poor support to their female partners in various maternal health issues are among the factors found to affect the maternal health information‐seeking behaviour of the women in the studied communities. Similar findings were also reported in a previous study by Mwangakala (2016). These findings, however, maybe because men, particularly in patriarchal settings in most African countries, play a dominant role in influencing maternal health choices in their families (Asa et al., 2017). The findings can also be attributed to the fact that for quite a long time most efforts to improve maternal health outcomes have been focused primarily on women thus leaving the men behind (Davis et al., 2016; Oyediran et al., 2002; Vouking et al., 2014). Therefore, it is apparent that in a situation like this, men are more likely to be left less informed about the importance of supporting their pregnant partners in various maternal health issues.

While appropriate and timely antenatal care is essential in improving maternal and child health as reported in various studies (Deo et al., 2015; Wilunda et al., 2017), findings from this study show that a majority of women in the studied communities fails to access them due to poor roads in their villages. It has been observed in this study that almost all the studied villages were characterised by poor roads, making it difficult for pregnant women to travel to other villages where health care facilities are available. These findings suggest that disparities in terms of access to health care still exist in most remote rural areas of Tanzania due to the inaccessibility of these services because of poor infrastructure. The findings in the present study are similar with those from previous studies (Sibiya et al., 2018; Wilunda et al., 2017) which also reported poor road infrastructure and poor means of transport as barriers to accessing health care in most remote rural areas of Africa.

This study has also revealed that low levels of education among women limit their ability to access and understand health information from different sources. Previous studies, particularly Malata and Chirwa (2011), have also acknowledged the role of education in influencing women's ability to seek, understand and appreciate maternal health information. Furthermore, consistent with findings from previous studies (Davies & Bath, 2002; Manyerere, 2015), this study has also found that multiple roles played by women affect their frequency of access to information. Findings from this study show that family responsibilities such as domestic chores negatively influence women's health information‐seeking behaviour. It has been noted, for instance, that because of the scarcity of water in some studied villages, many women spend most of their time fetching water from nearby villages. These activities deny them time to access maternal health care services and maternal health information in particular.

## Conclusion

The analysis of the information needs of the respondents has revealed that there is na continung information challenge on the quality of maternal health care between what is provided and what is needed by rural women at the community level. The fact that most of the women are interested in a range of maternal health information suggests that there are many unmet needs for this information among women in rural areas. This shortfall is a clear deprivation of chances to make informed decisions that c

an have a positive impact on these women’s maternal health. These findings, therefore, underline the need to provide women in rural areas with timely and relevant information to raise their awareness and thus empower them to make prompt and informed decisions about their quality maternal health. It is also apparent from the findings of this study that women are using informal sources of information to meet their various maternal health information needs. Dependence on such sources increases the chances of receiving unreliable information that may result in coming up with poor decisions. As such, the establishment of more health facilities with adequate skilled health care to lessen the distances women have to cover is of paramount importance in ensuring that women get access to relevant, reliable and adequate maternal health information. Additionally, there is also a need to offer more training to CHWs to empower them to assist in the provision of relevant and accurate maternal health information to women. This will help to lessen the effect of limited access to professional health care in rural and remote areas. The finding that some women in this study were not able to read health information contained in print sources calls for health practitioners to take into consideration the levels of education of women when presenting them with different types of maternal health information. It is also essential to strengthen efforts and strategies meant to improve men’s beliefs and attitudes towards maternal health. This can be achieved through health education campaigns geared towards motivating men to fully engage in various maternal health issues so that they can support their pregnant partners. The findings of this study have practical implications for the formulation of strategies for providing maternal health information to women of reproductive age in rural areas. A better understanding of the information needs and the challenges the women face when seeking the information will help health practitioners and other health information experts in designing a health information delivery system that will consider the rural environment and the information‐seeking behaviour of women in these areas.

## Conflict of interest

The author declares no conflict of interest.

## References

[hir12329-bib-0001] Aborigo, R. A. , Moyer, C. A. , Gupta, M. , Adongo, P. B. , Williams, J. , Hodgson, A. , … Engmann, C. M. (2014). Obstetric danger signs and factors affecting health‐seeking behaviour among the Kassena‐Nankani of Northern Ghana: A qualitative study. African Journal of Reproductive Health, 18(3), 78–86. 25438512

[hir12329-bib-0002] Afnan‐Holmes, H. , Rusibamayila, N. , Makuwani, A. , Msemo, G. , Kerber, K. , Levira, F. , … Hussein, A. (2015). Tanzania’s countdown to 2015: An analysis of two decades of progress and gaps for reproductive, maternal, newborn, and child health, to inform priorities for post‐2015. The Lancet Global Health, 3(7), e396–e409. 10.1016/s2214-109x(15)00059-5 26087986

[hir12329-bib-0003] Asa, S. S. , Kupoluyi, J. A. , Oyinloye, B. O. , & Titilayo, A. (2017). Multivariate analysis of household decision making, contraceptive use and fertility behaviour among ever‐married men in Nigeria. African Research Review, 11(4), 74. 10.4314/afrrev.v11i4.7

[hir12329-bib-0004] Babbie, E. (2008). The basics of social research, 4th ed.. Belmont: Thomson Wadsworth.

[hir12329-bib-0005] Bakar, R. R. , Mmbaga, B. T. , Nielsen, B. B. , & Manongi, R. N. (2019). Awareness of danger signs during pregnancy and post‐delivery period among women of reproductive age in Unguja Island, Zanzibar: A qualitative study. African Journal of Reproductive Health, 23(1), 27–36. 10.29063/ajrh2019/v23i1.3 31034169

[hir12329-bib-0006] Black, R. E. , Laxminarayan, R. , Temmerman, M. , & Walker, N. (2016). In Reproductive, maternal, newborn, and child health: Key messages of this volume. Reproductive, maternal, newborn, and child health: disease control priorities., (2nd Vol., 3rd ed.). Washington, DC: The International Bank for Reconstruction and Development / The World Bank. https://www.ncbi.nlm.nih.gov/books/NBK361926/ 27227235

[hir12329-bib-0007] Collins, C. (2007). The discrepancy between the information pregnant women expect and receive in Ireland and the lost opportunity for health promotion and education. International Journal of Health Promotion and Education, 45(2), 61–66. 10.1080/14635240.2007.10708102

[hir12329-bib-0008] Das, A. (2013). Information‐seeking among pregnant women: a mixed method approach. *ProQuest Dissertations and Theses*, Florida: Florida State University. Retrieved from https://diginole.lib.fsu.edu/islandora/object/fsu%3A183695

[hir12329-bib-0009] Davies, M. M. , & Bath, P. A. (2002). Interpersonal sources of health and maternity information for Somali women living in the UK: Information‐seeking and evaluation. Journal of Documentation, 58(3), 302–318. 10.1108/00220410210425629

[hir12329-bib-0010] Davis, J. , Vyankandondera, J. , Luchters, S. , Simon, D. , & Holmes, W. (2016). Male involvement in reproductive, maternal and child health: A qualitative study of policymaker and practitioner perspectives in the pacific. Reproductive Health, 13(1), 1–11. 10.1186/s12978-016-0184-2 27423461 PMC4947267

[hir12329-bib-0011] Deo, K. K. , Paudel, Y. R. , Khatri, R. B. , Bhaskar, R. K. , Paudel, R. , Mehata, S. , & Wagle, R. R. (2015). Barriers to utilization of antenatal care services in Eastern Nepal. Frontiers in Public Health, 3, 1–7. 10.3389/fpubh.2015.00197 26322302 PMC4536371

[hir12329-bib-0012] DRC (2014).Dodoma Regional Report presented to the President of the United Republic of Tanzania Hon. Dr. Jakaya Mrisho Kikwete, Dodoma: Dodoma Regional Commission.

[hir12329-bib-0013] Greene, M. E. , Robles, O. J. , Bathala, S. , & Risi, L. H. (2013). Delivering success : scaling up solutions for maternal health, Washington DC: Wilson Center. https://www.wilsoncenter.org/sites/default/files/media/documents/publication/DeliveringSuccess.pdf

[hir12329-bib-0014] Hanson, C. , Cox, J. , Mbaruku, G. , Manzi, F. , Gabrysch, S. , Schellenberg, D. , … Schellenberg, J. (2015). Maternal mortality and distance to facility‐based obstetric care in rural southern tanzania: a secondary analysis of cross‐sectional census data in 226 000 Households. The Lancet Global Health, 3(7), e387–e395. 10.1016/S2214-109X(15)00048-0 26004775

[hir12329-bib-0015] Kabakyenga, J. K. , Östergren, P. O. , Turyakira, E. , & Pettersson, K. O. (2011). Knowledge of obstetric danger signs and birth preparedness practices among women in Rural Uganda. Reproductive Health, 8(1), 33. 10.1186/1742-4755-8-33. 22087791 PMC3231972

[hir12329-bib-0016] Killewo, J. , Anwar, I. , Bashir, I. , Yunus, M. , & Chakraborty, J. (2006). Perceived delay in healthcare‐seeking for episodes of serious illness and its implications for safe motherhood interventions in Rural Bangladesh. Journal of Health, Population, and Nutrition, 24(4), 403–412. PMC3001144 17591337

[hir12329-bib-0017] Laddunuri, M. M. (2013). Maternal mortality in rural areas of Dodoma region, Tanzania: A qualitative study. International Journal of Caring Sciences, 6(2), 236–242. Retrieved from http://search.ebscohost.com/login.aspx?direct=true&db=cin20&AN=104149870&site=ehost-live

[hir12329-bib-0018] Malata, A. , & Chirwa, E. (2011). Childbirth information feeds for first time malawian mothers who attended antenatal clinics. Malawi Medical Journal, 23(2), 42–46. 10.4314/mmj.v23i2.70747 PMC3627698 23074811

[hir12329-bib-0019] Manyerere, J. J. (2015). Information behavior of rural women involved in small and medium enterprises (SMEs) in Chamwino and Manyoni Districts of Central Tanzania, Pietermartzburg: University of KwaZulu‐Natal. Retrieved from https://pdfs.semanticscholar.org/eee0/c0590cd9ea3dd0744cdd942c6c65b524e0c7.pdf

[hir12329-bib-0020] Mboera, B. E. , Genjamin, K. , Mayala, K. P. , Senkoro, S. M. , & Magesa, A. Y. K. (2008). Mosquito net coverage and utilisation for malaria control in Tanzania. African Health Sciences, 8(2), 114–119. 19357761 PMC2584322

[hir12329-bib-0021] MoHCDGEC (2017). Tanzania National Health Policy. Dar es Salaam: Ministry of Health, Community Development, Gender, Elderly and Children (MoHCDGEC).

[hir12329-bib-0022] MoHCDGEC, MoH, NBS, OCGS, & ICF (2016). Tanzania Demographic and Health Survey and Malaria Indicator Survey (THDS‐MIS) 2015‐16. Dar es Salaam, Tanzania, and Rockville, Maryland, USA: MoHCDGEC, MoH, NBS, OCGS, and ICF.

[hir12329-bib-0023] MoHSW (2008). The National Road Map Strategic Plan to accelerate reduction of maternal, newborn and child deaths in Tanzania 2008–2015. Dar es Salaam: Ministry of Health and Social Welfare.

[hir12329-bib-0024] MoHSW (2009). Health Sector Strategic Plan III : July 2009 – June 2015: partnership for delivering the MDGs. Dar es Salaam: Ministry of Health and Social Welfare.

[hir12329-bib-0025] MoHSW (2014). The National Road Map Strategic Plan to accelerate reduction of maternal, newborn and child deaths in Tanzania 2008–2015: Sharpened One Plan. Dar es Salaam: Ministry of Health and Social Welfare.

[hir12329-bib-0026] Mwangakala, H. (2016). Pregnant women’s access to maternal health information and its’ impact on healthcare utilization behavior in rural Tanzania. London: Loughborough University.

[hir12329-bib-0027] National Bureau of Statistics & Office of Chief Government Statistics (2015). Mortality and health. Dar es Salaam: National Bureau of Statistics.

[hir12329-bib-0028] Nieburg, P. (2012). Improving maternal mortality and other aspects of women’s health: The United States’ global role. Washington DC: Center for Strategic and International Studies. Retrieved from https://csis-website-prod.s3.amazonaws.com/s3fs-public/legacy_files/files/publication/121003_Nieburg_MaternalMortality_Web.pdf

[hir12329-bib-0029] Oyediran, K. A. , Ishola, G. P. , & Feyisetan, B. J. (2002). Factors affecting ever‐married men’s contraceptive knowledge and use in Nigeria. Journal of Biosocial Science, 34(4), 497–510. 10.1017/S0021932002004972 12395865

[hir12329-bib-0030] Pembe, A. B. , Urassa, D. P. , Carlstedt, A. , Lindmark, G. , Nyström, L. , & Darj, E. (2009). Rural Tanzanian women’s awareness of danger signs of obstetric complications. BMC Pregnancy and Childbirth, 9, 1–8. 10.1186/1471-2393-9-12 19323836 PMC2667432

[hir12329-bib-0031] Shemdoe, R. S. (2011). Tracking effective indigenous adaptation strategies on impacts of climate variability on food security and health of subsistence farmers in Tanzania. In African Technology Policy Studies Network: Working Paper Series. Nairobi: African Technology Policy Studies Network.

[hir12329-bib-0032] Shieh, C. , Broome, M. E. , & Stump, T. E. (2010). Factors associated with health information‐seeking in low‐income pregnant women. Women and Health, 50(5), 426–442. 10.1080/03630242.2010.506152 20853218

[hir12329-bib-0033] Shija, A. E. , Msovela, J. , & Mboera, L. E. G. (2011). Maternal health in fifty years of Tanzania independence: Challenges and opportunities of reducing maternal mortality. Tanzania Journal of Health Research, 13(5 Suppl), 1–15. 10.4314/thrb.v13i5 26591990

[hir12329-bib-0034] Sibiya, M. N. , Ngxongo, T. S. P. , & Bhengu, T. J. (2018). Access and utilisation of antenatal care services in a rural community of eThekwini district in KwaZulu‐Natal. International Journal of Africa Nursing Sciences, 8, 1–7. 10.1016/j.ijans.2018.01.002

[hir12329-bib-0035] Silali, M. , & Owino, D. (2016). Factors influencing accessibility of maternal & child health information on reproductive health practices among rural women in Kenya. Family Medicine & Medical Science Research, 05(01), 1–7. 10.4172/2327-4972.1000198

[hir12329-bib-0036] Song, H. , Cramer, E. M. , McRoy, S. , & May, A. (2013). Information needs, seeking behaviors, and support among low‐income expectant women. Women and Health, 53(8), 824–842. 10.1080/03630242.2013.831019 24215275

[hir12329-bib-0037] United Nations (2015). Transforming our world: the 2030 Agenda for sustainable development: resolution adapted by the general assembly on 25 September 2015. Retrieved from http://www.un.org/ga/search/view_doc.asp?symbol=A/RES/70/1&Lang=E

[hir12329-bib-0038] UNICEF (2012). Fact sheet maternal and child health: Tanzania's progress in maternal and child health. New York: UNICEF. Retrieved from http://www.unicef.org/tanzania/Fact_sheet_for_maternal_and_child_health_2012.pdf

[hir12329-bib-0039] Urassa, D. P. , Pembe, A. B. , & Mganga, F. (2012). Birth preparedness and complication readiness among women in mpwapwa district, Tanzania. Tanzania Journal of Health Research, 14(1), 1–7. 26591746 10.4314/thrb.v14i1.8

[hir12329-bib-0040] United Republic of Tanzania (2005). National Strategy for Growth and Reduction of Poverty (NSGRP) I. Dar es Salaam: Ministry of Finance and Economic Affairs.

[hir12329-bib-0041] United Republic of Tanzania (2010). National Strategy for Growth and Reduction of Poverty II. Dar es Salaam: Ministry of Finance and Economic Affairs. Retrieved from https://www.international.gc.ca/development-developpement/assets/pdfs/countries-pays/NATIONAL-STRATEGY-FOR-GROWTH-AND-REDUCTION-OF-POVERTY-TANZANIA.PDF

[hir12329-bib-0042] United Republic of Tanzania (2013). National post‐MDGs development Agenda consultations: The National Synthesis Report. Dar es Salaam: Economic and Social Research Foundation (ESRF).

[hir12329-bib-0043] Vouking, M. Z. , Evina, C. D. , & Tadenfok, C. N. (2014). Male involvement in family planning decision making in sub‐Saharan Africa: What the evidence suggests. Pan African Medical Journal, 19, 1–5. 10.11604/pamj.2014.19.349.5090 10.11604/pamj.2014.19.349.5090 PMC4406389 25922638

[hir12329-bib-0044] WHO (2017). Trends in maternal mortality: 2000 to 2017: Estimates by WHO, UNICEF, UNFPA, World Bank Group and the United Nations Population Division. Geneva: World Health Organization.

[hir12329-bib-0045] WHO , UNICEF , WORLD BANK , UNFPA , & UN (2014). Trends in maternal mortality : 1990 to 2013 estimates by WHO, UNICEF, UNFPA, The World Bank and the United Nations Population Division (Vol. 32). Geneva: World Health Organization. https://apps.who.int/iris/bitstream/handle/10665/112682/9789241507226_eng.pdf;jsessionid=6392D9646884685DE0052DEBAF0395E1?sequence=2

[hir12329-bib-0046] Wilunda, C. , Scanagatta, C. , Putoto, G. , Montalbetti, F. , Segafredo, G. , Takahashi, R. , … Betrán, A. P. (2017). Barriers to utilisation of antenatal care services in south Sudan: A qualitative study in Rumbek North County. Reproductive Health, 14(1), 1–10. 10.1186/s12978-017-0327-0 28532513 PMC5440928

